# Impact of Aging and a High-Fat Diet on Adipose-Tissue-Derived Extracellular Vesicle miRNA Profiles in Mice

**DOI:** 10.3390/biomedicines12010100

**Published:** 2024-01-03

**Authors:** Young-Eun Cho, Shaoshuai Chen, Keith Crouch, Joseph Yun, Aloysius Klingelhutz

**Affiliations:** 1 College of Nursing, The University of Iowa, 50 Newton Road, Iowa City, IA 52242, USA; 2 Predictiv Care, Inc., 800 West El Camino Real, Mountain View, CA 94040, USA; 3 Department of Microbiology and Immunology, College of Medicine, The University of Iowa, 51 Newton Road, Iowa City, IA 52242, USA

**Keywords:** aging, obesity, adipose tissue, extracellular vesicles, miRNAs

## Abstract

Background: Middle-aged adults have the highest obesity rates, leading to significant health complications in later years. Obesity triggers the release of altered molecules, including extracellular vesicles (EVs) from excess adipose tissue (AT), contributing to various health complications. In this study, we assessed the effects of age and a high-fat diet on AT-derived EV miRNA profiles to understand their potential roles in aging and obesity. Method: C57BL/6 male mice were subjected to a normal chow diet (NCD) or a high-fat diet (HFD) for either 10–12 weeks (young mice, *n* = 10) or 50–61 weeks (middle-aged mice, *n* = 12). After evaluating metabolic characteristics, peri-gonadal white AT was isolated and cultured to obtain EVs. AT-derived EV miRNAs were profiled using a NanoString miRNA panel (*n* = 599). Results: Middle-aged mice exhibited obesity regardless of diet. Young mice fed an HFD showed similar metabolic traits to middle-aged mice. In the NCD group, 131 differentially expressed miRNAs (DE-miRNAs) emerged in middle-aged mice compared to young mice, including miR-21, miR-148a, and miR-29a, associated with cancer, neuro/psychological disorders, and reproductive diseases. In the HFD group, 55 DE-miRNAs were revealed in middle-aged mice compared to young mice. These miRNAs were associated with significantly suppressed IGF1R activity. Conclusion: This study demonstrates the potential significant impact of miRNAs of AT EVs on aging- and obesity-related diseases.

## 1. Introduction

The rate of obesity has been growing continuously worldwide and is a significant global health challenge. In the United States (US), nearly one in three adults is overweight, and over two in five adults are classified as obese [1]. Among them, middle-aged adults (40–59 years old) exhibit the highest prevalence of obesity, with rates as high as 44.8%, and middle-aged men have a higher obesity rate than women [2,3]. People gain weight as they get older due to multiple factors, including a slowdown in metabolism, decreased muscle mass, and hormonal changes [4]. Furthermore, people in the US persistently consume a high-fat diet, which increases the risk of middle-aged adults in the US being obese [5]. Overweight and obese middle-aged adults are prone to experience more serious health complications when they are older than people who have a normal BMI in middle age, which include not only cardiovascular diseases and metabolic disorders, but also neurodegenerative diseases and cancers [2,6,7,8,9]. To mitigate these detrimental health outcomes and increase life expectancy, it is crucial to comprehend the dynamics of obesity in middle-aged adults.

Adipose tissue (AT) serves as an active endocrine organ beyond its conventional perception as a passive energy storage depot. AT secretes a wide array of bioactive molecules, which mediate numerous physiological processes such as insulin sensitivity, inflammation, and lipid metabolisms [10]. In obesity, expanded white AT (WAT) produces aberrant proinflammatory adipokines that promote a chronic inflammatory condition, contributing to the pathogenesis of various obesity-associated complications [11]. Meanwhile, AT is also a vulnerable tissue in aging. From middle age, AT alteration is accelerated, and proinflammatory adipokines released from altered AT influence aging-related health complications [12,13].

WAT is the primary contributor to circulating EVs [14]. EVs carry various cargo molecules, including miRNAs, proteins, and lipids of donor cells, through the bloodstream or the lymphatic system, enabling long-distance communication among organs. In cases of obesity, expanded and altered WAT releases increased quantities of EVs containing altered molecules, playing a role in complications associated with obesity, including not only metabolic diseases but also neurological disorders and cancers [15,16,17]. In aging, however, the impact of aging EVs derived from WAT remains unexplored, although WAT alteration is also recognized as an early event in aging. Given the growing evidence demonstrating the critical role of AT in various diseases, modulated AT-derived EVs by aging are anticipated to influence aging-related health complications. Additionally, within the United States, the aging process is frequently concomitant with a high-fat diet, attributable to the prevailing dietary trends in the nation. Aging can be accelerated by a prolonged high-fat diet, potentially exerting a synergistic impact effect on AT alteration. Therefore, in this study, we investigated WAT-derived EV miRNAs using a diet-induced obese model of middle-aged and young mice. The findings from this research will contribute to a deeper understanding of the involvement of WAT-derived EVs in health complications linked to aging and obesity, potentially uncovering therapeutic targets for intervention.

## 2. Methods

### 2.1. Animals

This study was performed in strict accordance with all applicable federal and institutional policies. The protocol was approved by the University of Iowa Animal Ethics Committee (Animal Protocol 1082418-004). C57BL/6J male mice (4–5 weeks old) were obtained from Jackson laboratory and assigned to either a normal chow diet (NCD) or a high-fat diet (60% fat, HFD, Research Diet, Inc., New Brunswick, NJ, USA) randomly. Mice were subjected to either diet for 10–12 weeks (young mice, *n* = 5 in each diet group) or 50–61 weeks (middle-aged mice, *n* = 6 in NCD and *n* = 4 in HFD), and euthanized at a mean age of 17 weeks old (young mice) and 56–67 weeks old (middle-aged mice). The number of mice was determined according to power analysis, indicating a target of 9.054 samples for each group of young and middle-aged mice.

### 2.2. Metabolic Characteristic Tests

The glucose tolerance test (GTT) was performed after overnight fasting (approximately 14 h). Glucose (2 mg of 10% glucose per body mass (kg)) was administered intraperitoneally, then blood glucose levels were measured from the tail-tip using a Care Touch glucometer (Future Diagnostics, Brooklyn, NY, USA) before and at 15, 30, 60, and 120 min after glucose administration. The insulin tolerance test (ITT) was performed after 8 h of fasting. Insulin (0.75 IU insulin (Novolin R Insulin, 100 UN/mL, Novo Nordisk Inc., Plainsboro Township, NJ, USA) per body mass (kg)) was administered intraperitoneally. Blood glucose levels were measured from the tail-tip using a Care Touch glucometer before and at 15, 30, 60, and 120 min after insulin administration. The time course of absolute blood glucose recorded during the ITT and the areas under the blood glucose curves (AUC) were used to evaluate insulin sensitivity. After completion of the test, the mice were returned to their home cage and given free access to food and water.

Fasting plasma insulin was measured from blood obtained during euthanasia using a mouse insulin kit (ALPCO Diagnostics, Salem, NH, USA). Absorbance at 450 nm was assessed via a microplate reader. The detailed procedure was followed according to the manufacturer’s instructions.

### 2.3. Adipose-Tissue-Derived Extracellular Vesicles

Peri-gonadal WAT was isolated from mice and minced in a culture dish into <1 mm^2^ size. Tissues were then cultured in culture media at 37 °C with 5% CO_2_. The culture media composition was DMEM with 10% exosome-free FBS and 50 µg/mL of gentamicin/amphotericin (ThermoFisher Scientific, Waltham, MA, USA). After 48 h, tissues were removed using a 100 µm cell strainer, and the culture medium was centrifuged at 3000× *g* for 15 min to remove cell debris and lipid layers. The strained medium was then filtered with a 0.22 µm syringe filter. EVs were extracted using Exoquick-TC (System Bioscience, Palo Alto, CA, USA). Detailed procedures for sample preparation were performed following the manufacturer’s instructions. The amount of EVs was measured with a Qubit™ Protein Assay (ThermoFisher Scientific). The size and concentration of EVs were analyzed using a nanoparticle tracking analysis (ViewSizer 3000, Horiba Instruments Inc., Tokyo, Japan). A general EV marker, Flotillin-1 (BD Bioscience, Franklin Lakes, NJ, USA), and adipocyte-derived EV markers, FABP4 (Santa Cruz Biotechnology, Inc., Dallas, TX, USA), were measured using Western blotting.

### 2.4. miRNA Profiling and Analysis

RNAs were isolated using a mirVana miRNA Isolation Kit (ThermoFisher Scientific) and cleaned and concentrated with an RNA Clean and Concentrator Kit (Zymo Research, Tustin, CA, USA). The total RNA concentration was adjusted to approximately 100 ng/µL in each sample. RNA samples were loaded on an nCounter^®^ mouse miRNA panel on an nCounter^®^ MAX Analysis System (NanoString, Seattle, WA, USA) following the manufacturer’s protocol.

Each sample’s raw data were normalized by mean values of housekeeping genes (*ACTB*, *B2M*, *GAPDH*, and *RPL19*) per each sample using nSolver version 4.0.70. A differential expression analysis was performed using DESeq2 version 1.38.3. For the *p*-value calculation, we used DESeq2 normalized expression values which account for the dispersion of each sample’s expressions. A differential expression analysis was performed using DESEQ2. We used the adjusted *p*-value of the False Discovery Rate (FDR) by Benjamini–Hochberg for multiple testing corrections. The cutoff value of the adjusted *p*-value was 0.05. The experiments were performed in batches, and the batch effects were controlled as a covariate in DESEQ2. 

### 2.5. Statistics

Metabolic characteristics between groups were compared using a Mann–Whitney U test or a two-way ANOVA with a statistical threshold of *p* = 0.05 using GraphPad Prism 10.0.2 (GraphPad Software, Inc., Boston, MA, USA). An Ingenuity Pathway Analysis (Qiagen, Hilden, Germany) was used to analyze network interactions among differentially expressed miRNAs and their target genes. All plots were generated using GraphPad Prism 10.0.2 (GraphPad Software, Inc.). 

## 3. Results

**Figure 1:**
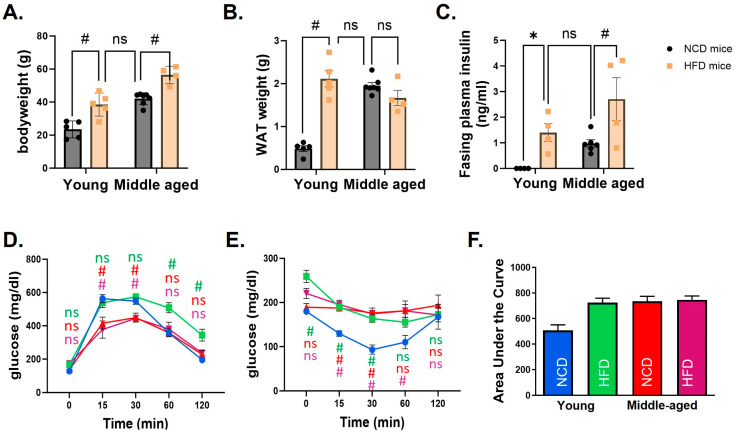
Metabolic characteristics of young and middle-aged mice fed a normal chow diet (NCD) or a high-fat diet (HFD). In both young and middle-aged mice fed an HFD, body weight (**A**) and white adipose tissue (WAT) weight (**B**) were significantly increased compared to mice fed an NCD. However, there was no difference between young mice fed an NCD and middle-aged mice fed an HFD. Fasting plasma insulin levels (**C**) revealed the same pattern. Glucose tolerance (**D**) and insulin tolerance tests (**E**) showed that all mice had impaired glucose control compared to young mice fed an NCD. A comparison of the area under the curve (AUC) of the insulin tolerance test clearly showed differences among groups (**F**). In (**D**,**E**), statistical significances were calculated at each time point based on young mice fed an NCD. The corresponding group colors represent the statistical results of each group compared to young mice fed an NCD in blue: green for young mice fed an HFD, red for middle-aged mice fed an NCD, and purple for middle-aged mice fed an HFD. * indicates *p* < 0.05, # indicates *p* < 0.01, and ns indicates no significance. Data are presented as means ± SE.

Body weight was significantly different in mice fed an HFD compared to an NCD in both age groups. However, the body weight of middle-aged mice fed an NCD was similar to that of the young mice fed an HFD. The weight of WAT showed the same pattern. While WAT weight was significantly higher in the HFD group compared to the NCD group in young mice, no statistically significant difference was observed in middle-aged mice (Figure 1A,B). Metabolic characteristics were compared among groups. A glucose tolerance test demonstrated that young mice fed an HFD had a delayed response after glucose injection compared to the NCD group. In middle-aged mice, blood glucose changes were not different between NCD and HFD groups. The increase in glucose level was significantly lower in middle-aged mice than in young mice. An insulin tolerance test demonstrated impaired insulin tolerance in all groups of mice except young mice fed an NCD (Figure 1C–F). 

**Figure 2:**
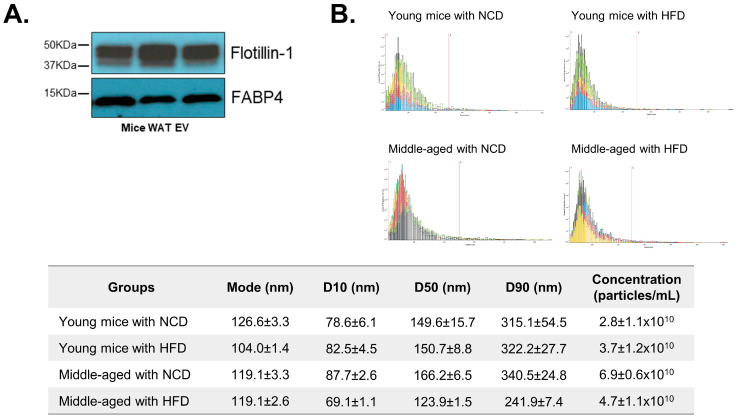
Characteristics of adipose-tissue-derived extracellular vesicles (AT EVs). The protein expression of an extracellular vesicle marker, Flotillin-1, and an adipose tissue marker, FABP4, was measured from AT EVs using Western blotting (**A**). The size and concentration of AT EVs were evaluated using a nanoparticle tracking analysis system. The *x*-axis of the graph shows the size range of the particle (0 to 1200 nm),and the red bar marks 500 nm. Each color of the graph indicates each sample tested (*n* = 3/each group). D10, D50 and D90 values mean percent undersize (**B**). Data are shown as means ± SE.

The characteristics of WAT-derived EVs were evaluated. The expression levels of Flotillin-1 (a general EV marker) and FABP4 (an adipocyte-derived EV marker) were measured to confirm EV characteristics (Figure 2A). A nanoparticle tracking analysis also showed that the size range of the majority of particles was within 100–300 nm (Figure 2B). Collectively, the results demonstrate that the obtained particles exhibit the distinctive characteristics of EVs derived from AT. 

**Figure 3:**
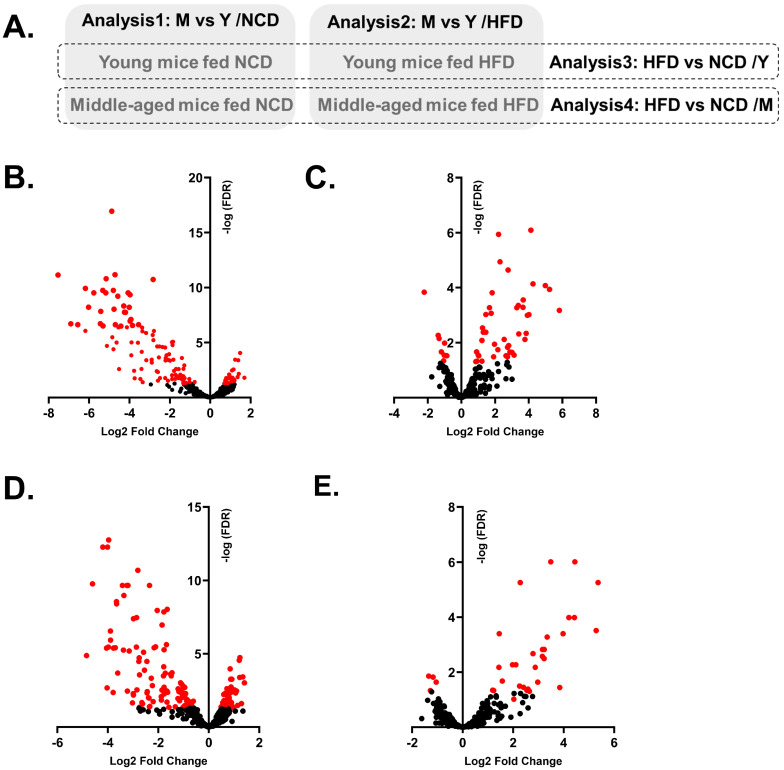
Four different DE-miRNA analyses and volcano plots of DE-miRNAs of each analysis. Schematic diagram displays four different combinations for analysis; Analysis1: M (middle-aged mice) vs. Y (Young mice)/NCD; Analysis2: M vs. Y/HFD; Analysis3: HFD vs. NCD/Y and Analysis4: HFD vs. NCD/M (**A**). Volcano plots demonstrate the expression patterns of DE- miRNAs in each analysis; Analysis1 (**B**), Analysis2 (**C**), Analysis3 (**D**), and Analysis4 (**E**). Dots in red indicate miRNAs with adjusted *p*-values < 0.05.

We then analyzed the WAT EV miRNAs among groups (Figure 3A). First, we analyzed the effect of aging on miRNA profiles in each diet group. DE-miRNAs in middle-aged mice (M) compared to young mice (Y) were identified in the NCD group (Analysis1: M vs. Y/NCD) and the HFD group (Analysis2: M vs. Y/HFD). Then, we compared the effect of an HFD on miRNA profiles in each age group. DE-miRNAs in the HFD group compared to the NCD group were identified in young mice (Analysis3: HFD vs. NCD/Y) and middle-aged mice (Analysis4: HFD vs. NCD/M). All DE-miRNAs of each analysis are listed in Supplementary File S1. 

Although middle-aged mice were not subjected to an HFD, their metabolic phenotypes were similar to obese mice. Therefore, in Analysis1: M vs. Y/NCD, miRNAs associated with both aging and obesity were identified as DE-miRNAs, including miR-126-3p, miR-151-5p, and miR-218. In fact, many DE-miRNAs in Analysis1: M vs. Y/NCD were overlapped with the DE-miRNAs identified from Analysis3: HFD vs. NCD/Y; out of 131 DE-miRNAs in Analysis1, 102 miRNAs overlapped with DE-miRNAs in Analysis3 with a similar expression direction. Most of the DE-miRNAs were downregulated in middle-aged mice and HFD mice (Supplementary Figure S1). Also, both Analysis1 and Analysis3 showed similar results from pathway analysis and enrichment analysis; an increase in activated Akt and FOXO1 pathways was identified as the main predicted pathways, and cancer, reproductive disease, and neuro/psychological diseases were identified as associated diseases with DE-miRNAs and their target genes (Table 1, Supplementary Figures S2 and S3). 

**Figure 4:**
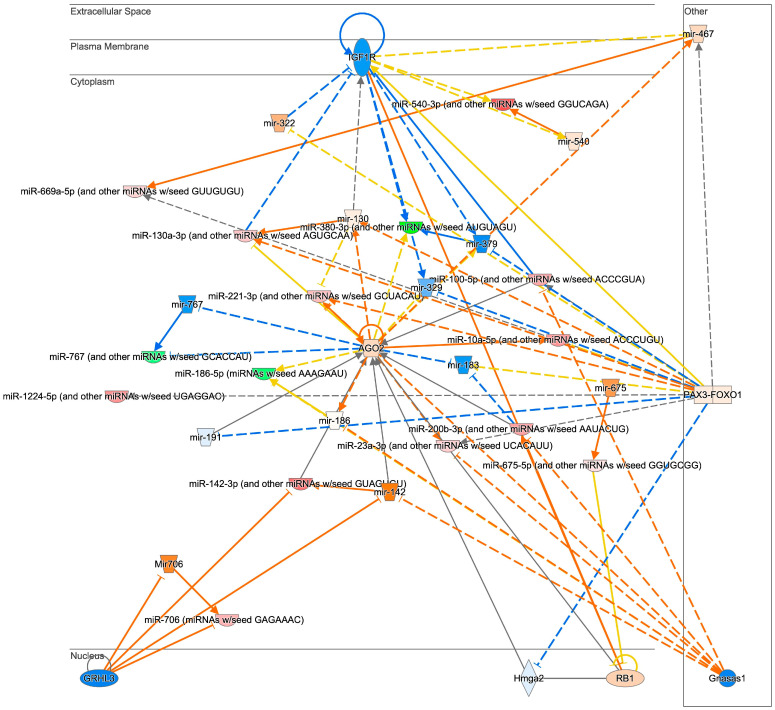
The anticipated pathways of DE-miRNAs in middle-aged mice fed an HFD compared to young mice fed an HFD (Analysis2: M vs. Y/HFD). Networking analysis of DE-miRNAs of middle-aged mice fed an HFD compared to young mice fed an HFD estimated that IGF1R is inhibited.

We also analyzed the effect of aging on miRNA profiles in HFD mice (Analysis2: M vs. Y/HFD). In contrast to the results of Analysis1: M vs. Y/NCD, the number of DE-miRNAs between groups was less (*n* = 52) and the changes were subtle. Also, most of the DE-miRNAs were upregulated in middle-aged mice (*n* = 44, Table 2, Figure 3C). From the pathway analysis, a strong inhibition of IGF1R was expected in middle-aged mice (Figure 4). Lastly, miRNA profiles of middle-aged mice were analyzed between HFD and NCD groups. In Analysis4: HFD vs. NCD/M, 33 DE-miRNAs were identified and 29 miRNAs were upregulated in the HFD group. A pathway analysis demonstrated a strong inhibition of Akt and CCND1 in the HFD group (Supplementary Figure S4).

## 4. Discussion

Growing evidence has suggested that WAT plays a vital role in not only obesity, but also aging-related pathophysiology as an endocrine organ. WAT releases significant amount of EVs, which are responsible for intra-organ crosstalk. miRNAs are major cargo molecules of EVs; therefore, it is critical to understand the altered WAT-derived EV miRNAs in aging and obesity. 

Aging and obesity share a similar pathological process and accelerate the processes of each other [12]. In this study, we also observed that middle-aged mice developed obesity regardless of diet and showed similar metabolic features to young obese mice. However, the AT weight of long-term HFD mice (HFD-fed middle-aged mice) was not different from that of the long-term NCD mice (NCD-fed middle-aged mice). While an HFD is usually associated with weight gain and an increase in WAT to store excess calories, there is evidence to suggest that prolonged exposure to an HFD can lead to changes in AT dynamics. It can trigger increased cell death in AT, particularly large adipocytes. This is known as hypertrophy-driven adipocyte death [18,19], which can outweigh the recruitment of new fat cells, leading to a net decrease in total cell numbers. Adipocyte apoptosis triggers an inflammatory response, leading to increased insulin resistance and type 2 diabetes development. The markedly elevated fasting plasma insulin levels of HFD-fed middle-aged mice compared to NCD-fed middle-aged mice may serve as indicative evidence of type 2 diabetes.

In middle-aged mice, not only age-related miRNAs, such as miR-34a and miR-15a, but also obesity-related miRNAs, such as miR-150 and miR-2141, were identified as altered. Due to the nature of WAT, altered miRNAs are also involved in metabolic regulation, such as insulin signaling pathways [15]. As EVs derived from WAT play a crucial role in circulating miRNAs, these modified miRNAs could significantly contribute to the development of metabolic disorders such as type 2 diabetes. In a recent study by Greco et al., circulating miRNAs associated with the development of type 2 diabetes in humans were identified [20]. They analyzed blood samples obtained from middle-aged people and identified significantly differentially expressed miRNAs in pre- and new-onset type 2 diabetic patients compared to controls. Despite species differences, several miRNAs, including miR-26b, miR-195, miR-100, miR-305, and miR-195, which were upregulated in young obese mice, were also elevated in pre- and new-onset type 2 diabetic patients. This indicates that altered AT-derived EV miRNAs in obesity are involved in the development of type 2 diabetes. However, the middle-aged mice exhibited a distinct pattern, possibly attributed to the fact that those mice fed both diets experienced an increase in body weight and demonstrated type 2 diabetic characteristics. 

Besides insulin signaling and metabolic regulation pathways, recent studies demonstrated that altered WAT is significantly associated with other pathological conditions [21]. Our results also suggest that altered AT EV miRNAs by obesity and aging are associated with cancers, neuro/psychiatric disorders, and reproductive diseases. DE-miRNAs altered by an HFD and aging, such as miR-126, miR-151, and miR-29a, have been identified as playing a crucial role in tumor progression, cancer growth, and metastasis [16,21]. DE-miRNAs such as miR-21, miR-148a-3p, miR-451 and miR-29 are associated with neurodegenerative diseases such as Alzheimer’s disease and Parkinson’s disease [22,23]. These miRNAs also influence puberty, sexual maturation, and fertility [24,25]. As such, our findings are aligned with previous studies demonstrating the critical role of the altered miRNA of WAT in aging- and obesity-related health complications, and also provide evidence of a potential underlying mechanism of how WAT might play a role. WAT-derived EVs are a major contributor to circulating miRNAs. EVs can transfer concentrated miRNAs of WAT to distant target organs while preventing potential degradation in circulation. Future studies need to reveal how these miRNAs work in inter-organ crosstalk, which can deepen our understanding of disease processes or development. 

Among miRNAs significantly altered in obese mice, some miRNAs, such as miR-21 and miR-148a, are known to be highly expressed in the WAT of obese individuals and animal models and regulate adipogenesis in WAT [26,27]. However, it is also reported that the level of circulating miR-21 is negatively correlated with body mass index, waist circumference, and insulin levels [28]. A recent preclinical study also reported that the oral treatment of miR-21 mimics inhibited increasing body weight and enhanced metabolic function [29]. Circulating miR-148a demonstrated a similar pattern, which was negatively correlated with body mass index [30]. In our study, we also observed that both miR-21 and miR-148a were markedly downregulated in AT-EVs of all mice groups that were obese. This discrepancy between WAT tissue and WAT-derived EVs might be elucidated by the selective EV cargo sorting mechanism. Among EV cargos, RNA sorting is not random; instead, the sorting process is highly specific [31,32]. It is suggested that miRNA loading can be controlled by miRNAs and specific endogenous target sequences [33]. However, the mechanism of how cells choose cargo for packaging into EV remains largely unknown. In particular, the potential beneficial or disease-contributing role of this process has not been explored. Unraveling this process will allow us to have a more comprehensive understanding of the role of WAT-derived EVs.

Although obese mice showed similar metabolic phenotypes and overlapped miRNAs regardless of age and diet, we anticipated that the miRNA profiles of middle-aged mice fed an HFD would specifically manifest deleterious features due to the potential synergistic effects of aging and long-term HFD consumption. Indeed, we found various significantly altered miRNAs in middle-aged mice fed an HFD compared to young mice fed an HFD (Analysis2). Several DE-miRNAs associated with both aging and obesity were identified: miR-150, miR-15b, miR-2141, miR-709, and miR-2132 [34,35]. Interestingly, a comprehensive network analysis revealed that DE-miRNAs and their target genes are associated with significant inhibition of insulin-like growth factor 1 receptor (IGF1R). Insulin and IGF1 act through highly homologous insulin receptor (INSR) and IGF1R expressed in AT [36]. In obese conditions, INSR expression in WAT is decreased, which is linked to the pathogenesis of type 2 diabetes. IGF1R plays a modest role in AT formation and function [36]; however, the expression of IGF1R in AT is not changed in obesity [37]. Meanwhile, IGF1R, expressed ubiquitously, is closely associated with aging [38]. Inhibited IGF1R activity has proven beneficial to lifespan by impeding various aging processes and relevant metabolic features, ultimately delaying the onset of age-related diseases [38,39,40]. Mice with low cardiac IGF1R levels demonstrated superior cardiac performance during aging and an increased maximum lifespan [41]. Brain IGF1R expression levels in mice negatively correlate with longevity [42]. In addition, recent research on IGF1R knock-out mice subjected to an HFD in their young and middle-aged stages revealed that middle-aged mice exhibited fewer metabolic changes compared to their insulin-resistant, hormone-disrupted young counterparts [43]. These findings support the idea that obesity in older age might confer benefits, with IGF1R potentially playing a role in this positive effect. This corresponds with the concept of the obesity paradox, suggesting that older individuals who are overweight or obese might encounter more favorable outcomes in specific diseases when compared to those who are of normal weight or underweight [44]. This concept is still controversial; however, recent studies examining the elderly population have revealed potential advantages associated with obesity among older adults [45,46]. Our results in middle-aged mice might support this concept, at least in the context of the protective role of WAT-derived EVs in later-life health. Further investigations are necessary to elucidate whether the IGF1R pathway is actually inhibited in WAT from HFD middle-aged mice compared to young mice, and how this inhibition might impact age- and obesity-related complications.

In this study, we tested a small number of male mice only. In general, there is a significant gender difference in weight change patterns and progression to obesity in both human and animal models; therefore, we deliberately confined our analysis to male mice only. This approach was chosen to mitigate potential complications arising from sex-related factors, allowing for a more streamlined examination of the specific parameters under investigation. In order to have a comprehensive understanding of the role of AT-derived EV studies, studies with female mice are needed. In addition, in our experimental setting, middle-aged mice fed an NCD developed obesity, as did mice subjected to an HFD. However, weight loss is also commonly seen in the middle-aged and older population. In order to attain a comprehensive understanding of the role of AT EVs, future studies should also include female mice and a weight loss group by aging.

## 5. Conclusions

This study has demonstrated the altered characteristics of WAT EV miRNAs by aging and diet. Aging and diet induce significant changes in EV miRNA profiles of AT. Our findings suggest that there might be a selective sorting mechanism of miRNAs in AT EVs, contributing to the disease progress or pathogenesis related to aging and obesity. Our findings also suggest that AT EVs in middle-aged mice fed an HFD might play a beneficial role by inhibiting IGF1R. Notably, this study contributes to understanding the role of AT EVs miRNAs at different ages and with different diets. 

## Figures and Tables

**Table 1:** Associated network functions of DE-miRNAs of Analysis1: M vs. Y/NCD and Analysis3: HFD vs. NCD/Y. DE-miRNAs (adjusted *p* < 0.05) in middle-aged compared to young mice fed an NCD (Analysis1) and HFD-fed young mice compared to NCD-fed young mice (Analysis3) demonstrate similar network functions.

		Associated Network Functions	Score
Analysis1	1	Cancer, Organismal Injury and Abnormalities, Reproductive System Disease	74
	2	Organismal Injury and Abnormalities, Reproductive System Disease, Neurological Disease	45
	3	Gene Expression, Organismal Injury and Abnormalities, Reproductive System Disease	31
	4	Neurological Disease, Organismal Injury and Abnormalities, Psychological Disorders	21
Analysis3	1	Cancer, Organismal Injury and Abnormalities, Reproductive System Disease	58
	2	Neurological Disease, Organismal Injury and Abnormalities, Psychological Disorders	41
	3	Gene Expression, Organismal Injury and Abnormalities, Reproductive system Disease	31
	4	Organismal Injury and Abnormalities, Reproductive System Disease, Gene Expression	31
	5	Gene Expression, Cancer, Organismal Injury and Abnormalities	28

**Table 2:** DE-miRNAs in middle-aged mice fed an HFD compared to young mice fed an HFD (Analysis2: M vs. Y/HFD).

	Fold Change	Adjusted *p*-Value
mmu-miR-150	5.822139065	0.000667
mmu-miR-2141	5.23792143	0.000116
mmu-miR-540-3p	4.98780634	8.39 × 10^−5^
mmu-miR-709	4.259419188	7.27 × 10^−5^
mmu-miR-2132	4.132817501	8.15 × 10^−7^
mmu-miR-142-3p	4.017360637	0.00096
mmu-miR-15b	3.92436743	0.001011
mmu-miR-1944	3.847249271	0.004598
mmu-let-7g	3.774034055	0.007608
mmu-miR-2138	3.677430123	0.00028
mmu-miR-2140	3.662169351	0.000518
mmu-miR-223	3.410221229	0.004813
mmu-miR-2145	3.386493889	0.000442
mmu-miR-2134	3.293558351	0.000537
mmu-miR-1224	3.128265323	0.028337
mmu-miR-10a	3.009093566	0.022669
mmu-let-7d	2.884564634	0.021905
mmu-miR-2146	2.807626526	0.01274
mmu-miR-2135	2.778755895	2.28 × 10^−5^
mmu-miR-1196	2.744215203	0.01475
mmu-miR-200c	2.743570896	0.032648
mmu-miR-2861	2.650268183	0.029966
mmu-miR-99a	2.541175848	0.007608
mmu-miR-714	2.298924059	1.15 × 10^−5^
mmu-miR-706	2.210911038	1.15 × 10^−6^
mmu-miR-710	2.167134724	0.018091
mmu-miR-221	1.969160296	0.011435
mmu-miR-191	1.9151175	0.032648
mmu-miR-804	1.827179043	0.000153
mmu-miR-300	1.769393482	0.000853
mmu-miR-130b	1.668842088	0.000537
mmu-miR-3471	1.45849	0.000947
mmu-miR-23b	1.452024959	0.004192
mmu-miR-1957	1.30088067	0.004192
mmu-miR-669a	1.257009852	0.002898
mmu-miR-2183	1.224234365	0.047275
mmu-miR-155	1.22423144	0.008295
mmu-miR-34c	1.22423144	0.008295
mmu-miR-543	1.22423144	0.008295
mmu-let-7e	1.029547522	0.029966
mmu-miR-1186	1.002690669	0.028302
mmu-miR-98	0.922521754	0.047275
mmu-miR-675-5p	0.905817452	0.021512
mmu-miR-1186b	0.853944548	0.048889
mmu-miR-471	−0.866500774	0.030061
mmu-miR-1193	−0.996865959	0.010413
mmu-miR-883a-3p	−1.013787058	0.028677
mmu-miR-494	−1.055986437	0.045656
mmu-miR-767	−1.189062126	0.021677
mmu-miR-1936	−1.320572595	0.007051
mmu-miR-186	−1.37489678	0.005413
mmu-miR-380-3p	−2.211211824	0.000146

## Data Availability

Available upon request.

## Supplementary Materials

The following supporting information can be downloaded at: https://www.mdpi.com/article/10.3390/biomedicines12010100/s1. File S1: Differentially expressed miRNAs in each analysis, Figure S1: Altered miRNAs in Analysis1: M vs. Y/NCD and Analysis3: HFD vs. NCD/Y, Figure S2: Top two anticipated pathways of DE-miRNAs in middle-aged mice fed NCD compared to young mice fed NCD (Analysis1: M vs. Y/NCD), Figure S3: Top two anticipated pathways of DE-miRNAs of young mice fed HFD compared to young mice fed NCD (Analysis3: HFD vs. NCD/Y), Figure S4: An anticipated pathway of DE-miRNAs in middle-aged mice fed HFD compared to middle-aged mice fed NCD (Analysis4: HFD vs. NCD/M).
