# Supplementary material for: Impact of Aging and a High-Fat Diet on Adipose-Tissue-Derived Extracellular Vesicle miRNA Profiles in Mice
Source: Biomedicines. 2024 Jan 3;12(1):100. doi: 10.3390/biomedicines12010100 (PMC10813715; doi:10.3390/biomedicines12010100)
Supplement: Supplementary file 1 [file biomedicines-12-00100-s001.zip › biomedicines-2728094-supplementary.pdf]

File S1: Differentially expressed miRNAs in each analysis
Analysis1_middle-aged_vs_young_for_NCD
Analysis2_middle-aged_vs_young_for_HFD
Analysis3_NCD_vs_HFD_for_young
Analysis4_NCD_vs_HFD_for_middle-aged

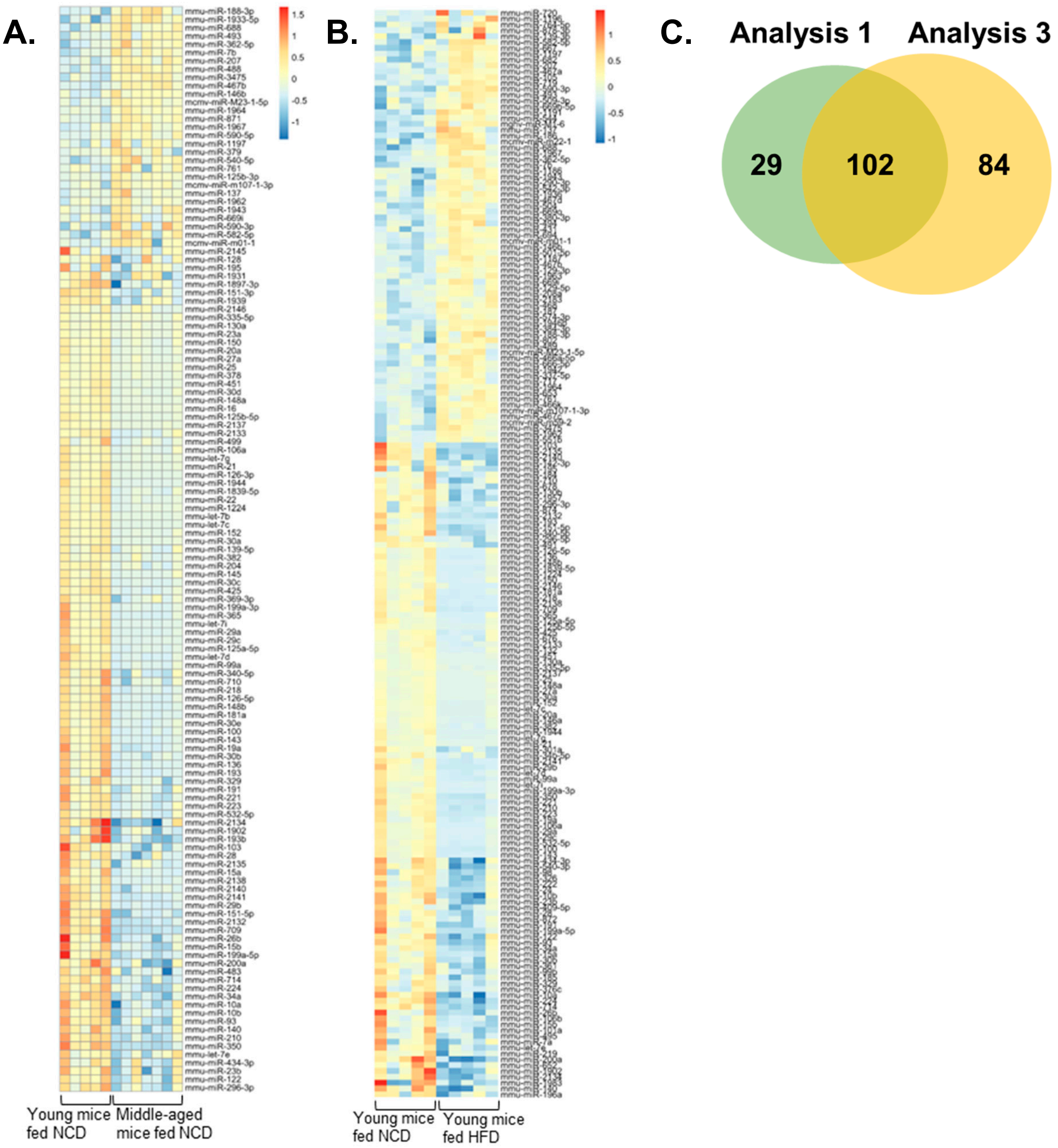

Figure S1. Altered miRNAs in Analysis1: M vs Y /NCD and Analysis3: HFD vs NCD /Y. Heatmaps demonstrate the expression patterns AT EV miRNA profiles (adjusted $p<0.05$) between young and middle-aged mice fed NCD **(A)** and young mice fed NCD and young mice fed HFD **(B)**. DE-miRNAs of each analysis shared common miRNAs **(C)**.

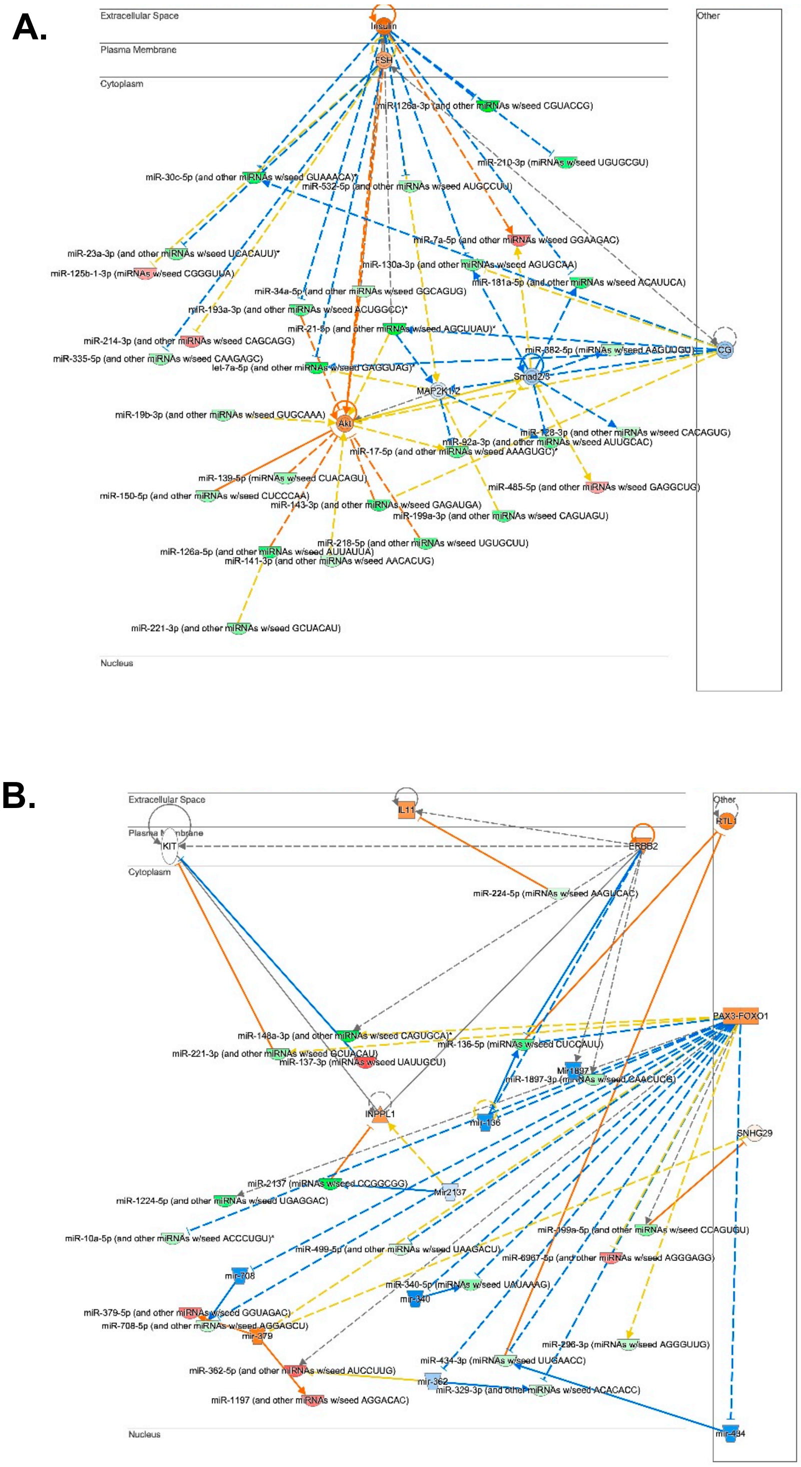

Figure S2. Top two anticipated pathways of DE-miRNAs in middle-aged mice fed NCD compared to young mice fed NCD (Analysis1: M vs Y /NCD).

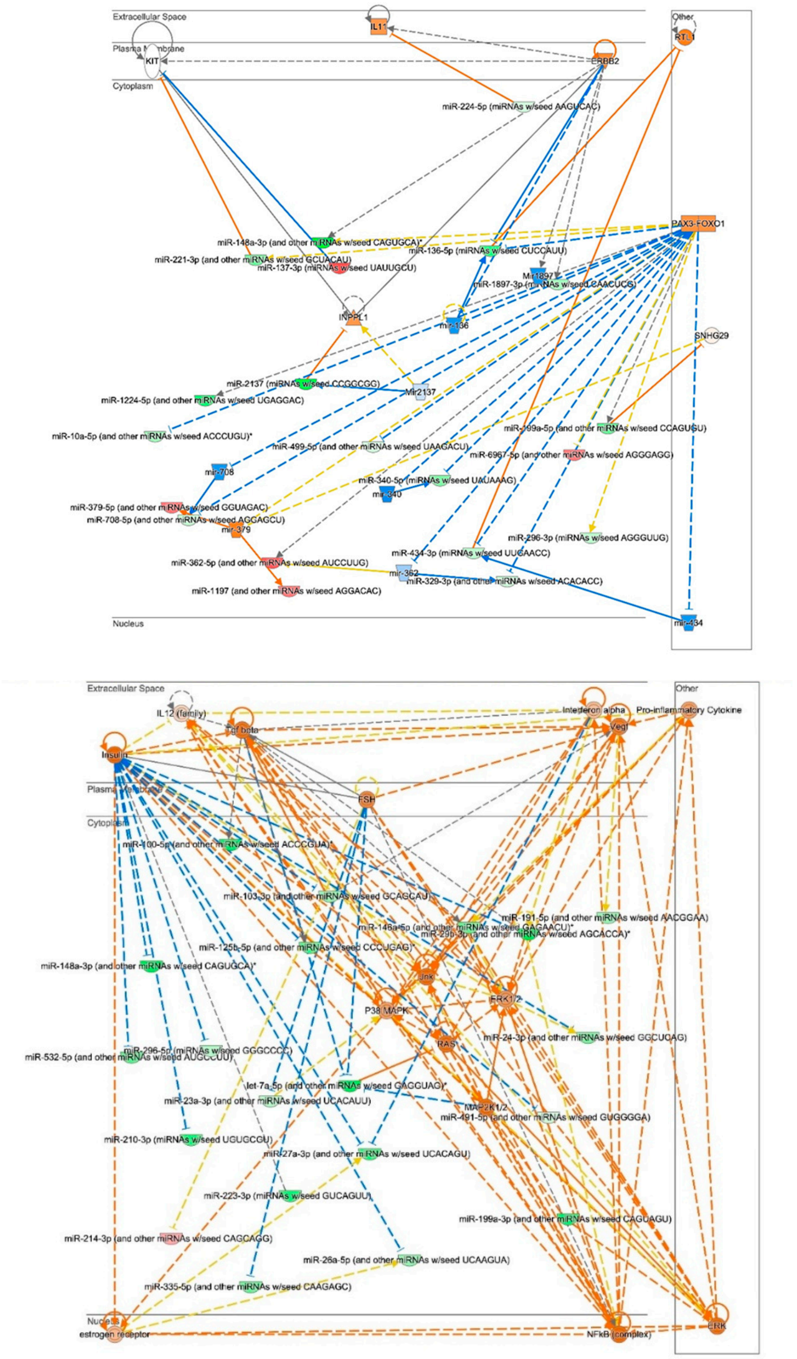

Figure S3. Top two anticipated pathways of DE-miRNAs of young mice fed HFD compared to young mice fed NCD (Analysis3: HFD vs NCD /Y).

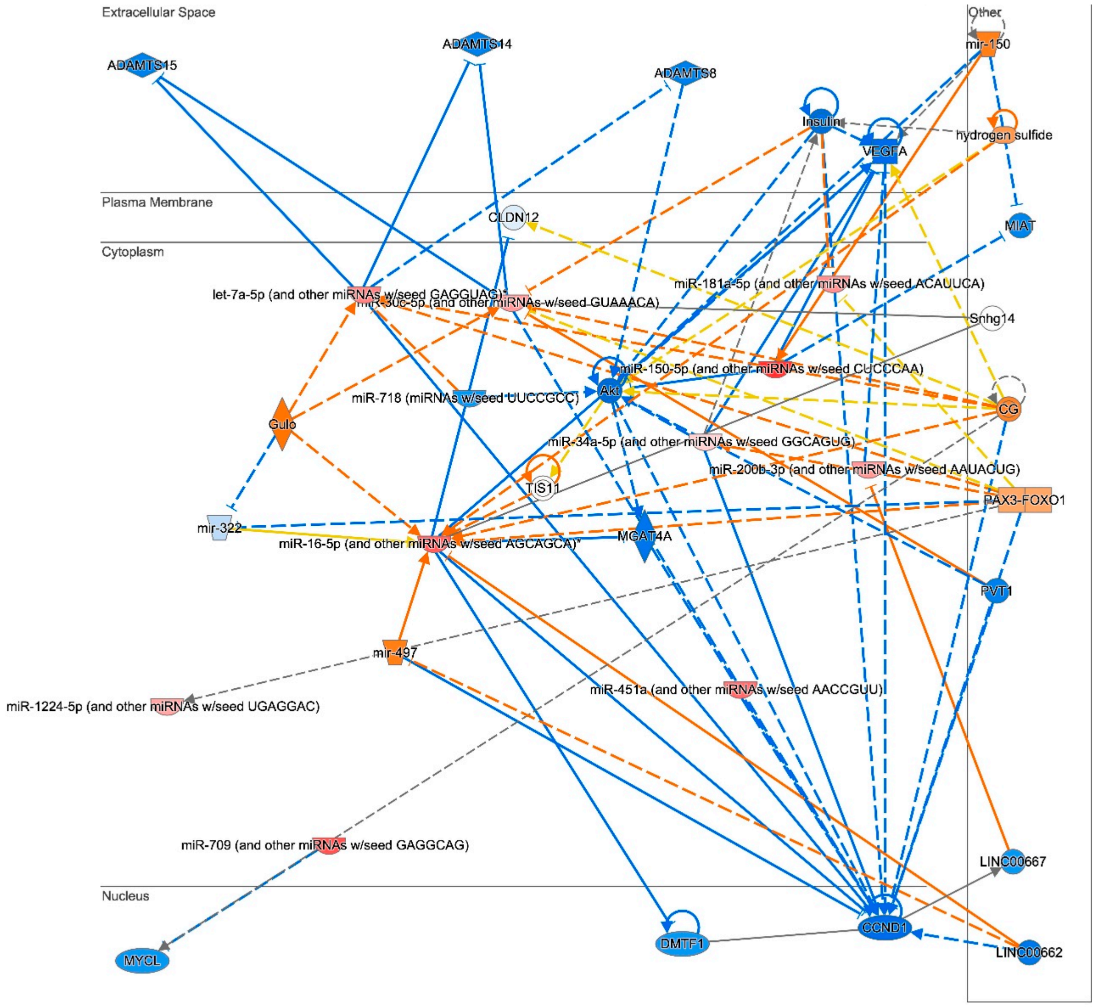

Figure S4. An anticipated pathway of DE-miRNAs in middle-aged mice fed HFD compared to middle-aged mice fed NCD (Analysis4: HFD vs NCD /M).
